# Non-lupus full-house nephropathy: a case series

**DOI:** 10.1590/2175-8239-JBN-2019-0242

**Published:** 2020-11-11

**Authors:** Márcia de Oliveira Silva, Patrick Vanttinny Vieira de Oliveira, Pedro Henrique Cavalcante Vale, Rinadja de Melo Cunha, Joyce Santos Lages, Dyego José de Araújo Brito, Natalino Salgado, Felipe Leite Guedes, Gyl Eanes Barros Silva, Ricardo Ferreira Santos

**Affiliations:** 1 Universidade Federal do Maranhão, Hospital Universitário Presidente Dutra, São Luís, MA, Brasil.; 2 Universidade Federal do Rio Grande do Norte, Hospital Universitário Onofre Lopes, Natal, RS, Brasil.; 3 Universidade Federal do Maranhão, Hospital Universitário Presidente Dutra, Laboratório de Imunofluorescência e Microscopia Eletrônica, São Luís, MA, Brasil.

**Keywords:** Lupus Erythematosus, Systemic, Lupus Nephritis, Fluorescent Antibody Technique., Lúpus Eritematoso Sistêmico, Nefrite Lúpica, Imunofluorescência.

## Abstract

Systemic lupus erythematosus (SLE) is a chronic multisystem autoimmune inflammatory disease. However, some patients may exhibit a histological pattern of kidney injury, with characteristics indistinguishable from lupus nephritis, but without presenting any extrarenal symptoms or serologies suggestive of SLE. Such involvement has recently been called non-lupus full-house nephropathy. The objective is to report a series of clinical cases referred to the Laboratory of the Federal University of Maranhão that received the diagnosis of "full-house" nephropathy unrelated to lupus, upon immunofluorescence and to discuss its evolution and outcomes. Non-lupus full-house nephropathy represents a diagnostic and therapeutic challenge, because it is a new entity, which still needs further studies and may be the initial manifestation of SLE, isolated manifestation of SLE or a new pathology unrelated to SLE.

## Resumo

O lúpus eritematoso sistêmico (LES) é uma doença inflamatória crônica autoimune multissistêmica. Alguns pacientes, contudo, podem exibir um padrão histológico de lesão renal, com características indistinguíveis da nefrite lúpica, porém sem apresentar quaisquer sintomas extrarrenais ou sorologias sugestivas de LES. Tal acometimento tem sido recentemente denominado nefropatia “full-house” não relacionada ao lúpus. O objetivo é relatar uma série de casos clínicos encaminhados ao Laboratório da Universidade Federal do Maranhão que receberam o diagnóstico de nefropatia “full-house” não relacionada ao lúpus à imunofluorescência e discutir sua evolução e desfechos. A nefropatia “full-house” não relacionada ao lúpus representa um desafio diagnóstico e terapêutico por ser uma entidade nova, que ainda necessita de maiores estudos e pode ser a manifestação inicial do LES, manifestação isolada do LES ou uma patologia nova não relacionada ao LES.

## Introduction

Systemic lupus erythematosus (SLE) is a chronic autoimmune inflammatory disease that involves different organs and systems, and exhibits a wide spectrum of clinical manifestations. Its incidence is higher in young women and its diagnosis was based on the criteria of the Systemic Lupus International Collaborating Clinics (SLICC), but the antinucleus factor (ANA) positivity has become paramount for the diagnosis of SLE, according to the new European League Against Rheumatism criteria (EULAR) and American College of Rheumatology (ACR).^1^^,^ 2 ^,^ 3 ^,^ 4 Lupus nephritis, defined by proteinuria greater than or equal to 500 mg/day or corresponding findings in renal biopsy, is one of its most serious and frequent complications and may be present in approximately 60% of cases.^2^^,^ 3 ^,^ 4 ^,^ 5 ^,^ 6

Histologically, the International Society of Nephrology (ISN) into six patterns, under light microscopy, classifies lupus nephritis: I. minimal mesangial, II. Proliferative mesangial, III. Focal proliferative, IV. Diffuse proliferative, V. membranous and VI. Sclerosing.^7^ Among the findings of indirect immunofluorescence (IIF), the following stand out: the positivity of glomerular deposits of IgG, IgA, IgM, C3 and C1q (“full-house” pattern), with a predominance of IgG over the other immunoglobulins, in addition to the presence of extraglomerular immune deposits in the basal tubular membranes, interstitium and blood vessels. Electron microscopy shows electrodense deposits in the mesangial, subendothelial and subepithelial regions, associated with the presence of tubular-reticular endothelial inclusions.^8^^,^ 9

Just a few patients present renal disorders as the only manifestation of the disease, with findings from renal biopsy (mainly from IIF) classically associated with SLE, but without presenting other diagnostic or serological criteria. This condition has been called non-lupus full-house nephropathy.^5^^,^ 8 ^,^ 10 ^,^ 11

The objective of this study is to report on a series of cases of non-lupus full-house nephropathy, a clinical entity that is still little described, especially in relation to its evolution and outcomes.

## Presentation of Clinical Cases

This study was prepared based on the analysis of three clinical cases referred to the Renal Pathology Laboratory of the University Hospital of the Federal University of Maranhão, a national reference for federal hospitals of the Brazilian Hospital Services Company (Ebserh). All patients had negative serologies for chronic infectious diseases (HIV, syphilis, hepatitis B and C) and at the time of the renal biopsy they had no diagnostic criteria for SLE (including negative ANA and normal serum supplements). None of the patients reported a personal or family history of autoimmune or kidney disease. In all cases, immunofluorescence revealed “full-house” nephritis (details of the histological and fluorescence patterns are available in Table 1).

**Table 1:** Summary of the cases

Case	Sex	Age (years)	Clinical manifestations	Creatininand proteinuria upon admission	Glomerular pattern in the Optic Microscopy	IIF	Treatment carried out	Clinical progress
1	Fem	44	Past of nephrotic syndrome 10 years ago. Edema recurrence 6 months ago. Normotensive. No hematuria.	Cr: 0.6 mg/dL Proteinuria: 6350 mg/24h	Glomerulonefrite membranosa 10 glomeruli with global GBM thickening and podocyte hypertrophy. Membranous glomerulonephritis	IgG, C3, C1q, light chains: 3+; IgA: 2+; IgM: 1+	Corticotherapy alone initially. Then, monthly intravenous cyclophosphamide was added for 6 months.	Partial response to corticosteroid therapy. 2nd month of cyclophosphamide use showed clinical remission (proteinuria 123mg/24h). Follow-up for 12.5 years.
2	Males	24	Edema of the lower limbs for 2 months, progressing to anasarca. Hypertensive. No hematuria.	Cr: 3.8 mg/dL Proteinuria: 11600 mg/24h	4 globally sclerosing glomeruli and 7 with mesangial expansion and thickening with GBM duplication of MBG	IgG: 3+; IgM, C3, C1q, light chains: 2+; IgA:1+	Oral corticosteroid, 1 mg / kg, initially. After 2 months, association with monthly cyclophosphamide, for 6 months.	No clinical response to the prescribed immunosuppression, evolved to RRT. Follow-up for 1 year.
3	Fem	24	Puerperium with recurrent edema that evolved to anasarca and oliguria. Past of pre-eclampsia. Hypertensive. No hematuria.	Cr: 0.4 mg/dL Proteinúria: 2345 mg/24h	Membranoproliferative glomerulonephritis 10 hypervolemic, hypercellular glomeruli with capillary lumen occlusion. 1 glomerulus with crescent. Diffuse proliferative glomerulonephritis, type III membranoproliferative	IgG, C3, C1q, light chains: 3+; IgM, IgA: 2+.	Started oral corticosteroids at 1 mg/kg. Azathioprine was associated, followed by sodium mycophenolate. Cyclophosphamide monthly a posteriori, for 6 months.	Refractory to oral corticosteroids, azathioprine and mycophenolate. Partial remission with cyclophosphamide. She presented a new flare with nephrotic proteinuria and progression to ESRD in another pregnancy, requiring RRT. Follow-up for 14 years.

Fem: female; Cr: serum creatinine; M.O.: optical microscopy; IIF: indirect immunofluorescence; CKD: chronic kidney disease; RRT: renal replacement therapy; GMB: glomerular basement membrane; ESRD: end-stage chronic kidney disease.

### Case 1

A 44-year-old brown woman with a history of nephrotic syndrome for ten years, abandoned treatment at the time. Six months ago, the edema returned, but she was normotensive and without hematuria. Renal function was normal and presented 6350 mg/24h proteinuria. Renal biopsy revealed secondary membranous glomerulonephritis.

She was initially treated with oral corticosteroid therapy, but did not show any remission of the disease (proteinuria persisted at 3538 mg/24h), and monthly pulse therapy with cyclophosphamide was started for six months. After the second cycle of cyclophosphamide, she presented remission of the disease, with recent proteinuria of 132 mg/24h.

### Case 2

A 24-year-old white male with a two-month history of lower limb edema associated with hypertension. There was progression to anasarca with a month of disease progression. At the time, he had creatinine 3.8 mg/dL and proteinuria of 11600 mg/24h. Renal biopsy showed a pattern of membranoproliferative glomerulonephritis and signs of chronic kidney disease.

Immunosuppressive treatment was started with prednisone 1.0 mg/kg empirically, but without improvement. He started cyclophosphamide pulse therapy in monthly cycles for six months. However, there was no satisfactory response, persisting in nephrotic syndrome (proteinuria 17.7 grams/24h) and renal dysfunction (creatinine 7.8 mg/dL). He was referred for hemodialysis.

### Case 3

A 24-year-old black woman with a history of pre-eclampsia in the first pregnancy, evolved in the puerperium with episodes of edema. After four years, she presented anasarca, associated with hypertension and oliguria. She had proteinuria (2345 mg/24h) and a normal renal function. Biopsy revealed diffuse proliferative glomerulonephritis.

She started treatment with prednisone 1.0 mg/kg and then associated with azathioprine, followed by sodium mycophenolate, but without a satisfactory response. Faced with persistent nephrotic proteinuria, she was submitted to the National Institutes of Health (NIH trials) regimen of 0.5 to 1 g/m^2^ of cyclophosphamide monthly for six months, achieving partial remission. In a new pregnancy after four years, there was worsening of proteinuria (8307 mg/24h), anasarca and renal dysfunction, with termination of pregnancy at the 28th week, and the need for dialysis therapy. In the postpartum period, she was followed up on an outpatient basis and, after three years, there was a progression from kidney disease to chronicity.

## Discussion

Our series presents three patients with a renal biopsy pathology result compatible with the “full-house” pattern at the IIF. The patients had a mean age of 30.6 years at the beginning of the renal impairment. In all cases, the clinical presentation was of an edemigenic syndrome, two were associated with hypertension and, in one case, the clinical manifestations started during pregnancy. Of the three patients, two had nephrotic proteinuria. Renal dysfunction was present in one of the cases at the beginning of the disease.

Regarding histological aspects, there was a predominance of proliferative forms (present in two of the cases presented), and one case in which a pattern of membranous nephropathy was identified. None of the cases presented hematuria in the regular urine test.

Likewise, no other clinical criteria for SLE were found in the patients described over the course of up to ten years, although biopsies were suggestive of lupus nephritis. Similarly, there are reports of patients who had biopsies compatible with lupus nephritis without clinical or serological manifestations; however, after a variable period, they developed them. Such a presentation could announce the appearance of lupus that is still incipient.^8^

Gianviti et al., in a series of cases, presented the report of three children with glomerulonephritis suggestive of SLE, but without clinical or serological evidence. After a ten-year follow-up, all of them were positive for ANA, and one of them developed a typical clinical picture of the disease, after six years of follow-up.^12^ Jones et al. presented a series of five adults with a “full-house” pattern in all cases. Although a patient had generalized arthralgia, no patient had criteria for SLE at any point during the average follow-up of two to three years.^13^ Wen et al. brought together 59 patients who presented non-lupus “full-house” nephropathy, in a literature review, and only seven patients developed clinical or serological evidence of SLE during the follow-up, which ranged from three months to ten years.^14^

Dias et al. presented 20 cases with non-lupus “full-house” nephropathy in an average follow-up of 64 months, and only 20% developed SLE; 15%, schistosomiasis; 5%, cryoglobulinemia; 5%, HIV; and the remaining 55% remained as an idiopathic form. In comparison with the group of lupus nephritis during follow-up, the group of non-lupus “full-house” nephropathy had higher initial proteinuria (8.40 g/day x 6.34 g/day, p = 0.04) and final (2.42 g/day x 0.80 g/day, p = 0.016), in addition to higher final serum creatinine (2.28 mg/dL x 1.10 mg/dL, p = 0.012).^15^

Other pathologies can manifest with standard full-house immunofluorescence and must be considered in the differential diagnosis of SLE: liver diseases, diabetes mellitus, primary glomerular diseases, C1q nephropathy, IgA nephropathy, infections (post-streptococcal glomerulonephritis, endocarditis), in addition of infection by the human immunodeficiency virus (HIV), hepatitis B or C, BK and CMV virus.^16^ None of the patients reported by us had any evidence of these diseases.

As for treatment, all three patients received a combination of corticosteroid therapy with cyclophosphamide in monthly pulses for six months, followed by maintenance therapy with immunosuppressants and low-dose corticosteroids. Both patients with proliferative forms progressed to renal replacement therapy. The patient with a histological pattern of membranous nephropathy had a favorable progress.

None of the patients in the present study met clinical criteria for SLE or had positive autoantibodies during the 1-14 year follow-up, which may have been brief for some of the cases. However, the involvement of autoantibodies, not routinely researched as causing the kidney injury described cannot be ruled out.^17^ Non-lupus “Full-house” nephropathy represents a diagnostic and therapeutic challenge because it is a new entity, which still needs greater studies and may be the initial manifestation of SLE, isolated manifestation of SLE or a new disease unrelated to SLE.
